# Anti-connective tissue growth factor (CTGF/CCN2) monoclonal antibody attenuates skin fibrosis in mice models of systemic sclerosis

**DOI:** 10.1186/s13075-017-1356-3

**Published:** 2017-06-13

**Authors:** Katsunari Makino, Tomoko Makino, Lukasz Stawski, Kenneth E. Lipson, Andrew Leask, Maria Trojanowska

**Affiliations:** 1 Arthritis Center, Boston University School of Medicine, 72 East Concord Street, E-5, Boston, MA 02118 USA; 2 FibroGen, Inc, San Francisco, CA USA; 3 Departments of Dentistry and Physiology and Pharmacology, Schulich School of Medicine and Dentistry, University of Western Ontario, Dental Sciences Building, London, ON Canada

**Keywords:** Angiotensin II, CTGF/CCN2, FG-3019, Fibrosis, Skin, Systemic sclerosis

## Abstract

**Background:**

Systemic sclerosis (SSc) is characterized by fibrosis of the skin and internal organs. Although the involvement of connective tissue growth factor (CTGF/CCN2) has been well-documented in SSc fibrosis, the therapeutic potential of targeting CTGF in SSc has not been fully investigated. Our aim was to examine the therapeutic potential of CTGF blockade in a preclinical model of SSc using two approaches: smooth muscle cell fibroblast-specific deletion of CTGF (CTGF knockout (KO)) or a human anti-CTGF monoclonal antibody, FG-3019.

**Methods:**

Angiotensin II (Ang II) was administered for 14 days by subcutaneous osmotic pump to CTGF KO or C57BL/6 J mice. FG-3019 was administered intraperitoneally three times per week for 2 weeks. Skin fibrosis was evaluated by histology and hydroxyproline assay. Immunohistochemistry staining was used for alpha smooth muscle actin (αSMA), platelet-derived growth factor receptor β (PDGFRβ), pSmad2, CD45, von Willebrand factor (vWF), and immunofluorescence staining was utilized for procollagen and Fsp1.

**Results:**

Ang II-induced skin fibrosis was mitigated in both CTGF KO and FG-3019-treated mice. The blockade of CTGF reduced the number of cells expressing PDGFRβ, procollagen, αSMA, pSmad2, CD45, and Fsp1 in the dermis. In addition, inhibition of CTGF attenuated vascular injury as measured by the presence of vWF-positive cells.

**Conclusions:**

Our data indicate that inhibition of CTGF signaling presents an attractive therapeutic approach in SSc.

## Background

Systemic sclerosis (SSc) is a multisystem connective tissue disorder that typically results in fibrosis of the skin and internal organs [1]. SSc is characterized by autoimmunity, inflammation, and widespread microvascular injury, leading to the activation of fibroblasts and excessive accumulation of extracellular matrix (ECM) proteins; however, the causes and the pathogenesis of this disease are not yet fully explained [2]. Accumulation of ECM proteins restricts normal function of the affected tissues and organs leading to high morbidity and mortality. Despite significant improvements in the management of SSc, effective disease modifying therapies are not yet available.

Connective tissue growth factor (CTGF, also known as CCN2), a member of the CCN family of matricellular proteins, is widely known as a hallmark of fibrosis in multiple tissues, including skin, heart, lung, liver, and kidney [3, 4]. A key study in support of the essential role of CTGF in fibrosis was published by Takehara and colleagues who showed that subcutaneous injection of transforming growth factor (TGF)-β and CTGF led to sustained fibrosis, while either factor alone failed to do so [5]. Further support was provided by a recent study that showed that fibroblast-specific ablation of CTGF inhibited development of dermal fibrosis in the bleomycin injection model [6]. The role of CTGF in fibrosis may not be limited to its function as a co-factor of TGF-β, but CTGF may also regulate other aspects of the fibrogenic process, consistent with its multifunctional nature [7, 8]. Accordingly, it has been suggested that CTGF may contribute to myofibroblast recruitment during bleomycin-induced skin fibrosis [6]. Transgenic mice overexpressing CTGF develop skin fibrosis and microvascular abnormalities [9], and loss of CTGF results in a reduction of bleomycin-induced skin fibrosis [6].

Accumulating reports show that CTGF is highly expressed in SSc [10]. Elevated levels of CTGF have been identified in fibrotic skin and serum from patients with SSc, and have been correlated with the severity of skin and lung fibrosis [11, 12]. Elevated CTGF protein expression has been observed in fibroblasts in affected skin in SSc [13]. Furthermore, N-terminal cleavage products of CTGF have been identified in interstitial fluid in the skin of patients with SSc [14].

Given the key role of CTGF in the development of skin fibrosis in SSc, the goal of this study was to evaluate the efficacy of FG-3019 as a potential therapeutic agent for SSc using a murine model of angiotensin II (Ang II)-induced skin fibrosis [15–17]. FG-3019 is a fully human monoclonal antibody specific for CTGF [18]. Clinically, FG-3019 is being evaluated for treatment of idiopathic pulmonary fibrosis, pancreatic cancer, and Duchenne muscular dystrophy [19–21]. In parallel studies, we employed mice with smooth muscle cell fibroblast-specific deletion of CTGF to assess the contribution of CTGF to the process of skin fibrosis in the Ang II model [6]. Together, these studies showed that FG-3019 was comparable to genetic CTGF deletion in attenuating skin fibrosis. The results of this study support the use of FG-3019 as therapy for skin fibrosis.

## Methods

### Reagents

A fully human IgG1kappa monoclonal antibody recognizing domain 2 of human and rodent CTGF (FG-3019) and whole human IgG control antibody were obtained from Fibrogen (San Francisco, CA, USA).

### Mice

Smooth muscle cell fibroblast-specific CTGF knockout (KO) mice were generated as described [6]. In brief, mice with CTGF flanked by loxP sites were crossed with mice containing a Cre recombinase gene located downstream of a collagen α2 (I) promoter enhancer that confers fibroblast-specific gene expression. The expression of Cre is dependent on the presence of tamoxifen. Tamoxifen was diluted in corn oil to 10 mg/ml. Three-week-old mice were given intraperitoneal injections of the tamoxifen suspension (100 μl of 10 mg/ml) over 10 days. Three weeks later, the mice were used for further studies. We used mice homozygous for CTGF gene flanked by loxP sites and heterozygous for Cre as CTGF KO mice. Littermate mice homozygous for loxP-flanked CTGF that were wild for Cre (non Cre) were used as control mice. C57BL/6 J mice for FG-3019 injection were purchased from the Jackson Laboratory (Bar Harbor, ME, USA). All mice experiments were performed in accordance with the National Institutes of Health and institutional guidelines for animal care, and approved by the Committee on the Ethics of Animal Experiments of the Boston University (Protocol AN-15037).

### Cell culture and immunoblotting

Mouse dermal fibroblasts were obtained from skin from the backs of male CTGF KO and control littermate mice. Fibroblasts between the third and fifth sub passages were used for experiments. Mouse dermal fibroblasts were lysed and subjected to immunoblotting, as described previously [22]. Primary antibodies used were (1:1000): CTGF from Santa Cruz Biotechnology (Dallas, TX, USA) and β-actin from Sigma (St. Louis, MO, USA).

### Ang II-induced dermal fibrosis

Alzet osmotic miniature pumps (Model 1002, DURECT, Cupertino, CA, USA) delivering Ang II (EMD Millipore, Billerica, MA, USA) at a rate of 1000 ng/kg/min (pressor dose) or PBS, were implanted subcutaneously on the back of 8-week-old mice, as described previously [17]. After 2 weeks, mice were euthanized and the skin surrounding the pump outlet was collected. FG-3019 (25 mg/kg) or control IgG (25 mg/kg) was administered intraperitoneally three times per week for 2 weeks, after the osmotic pump was installed.

### Histologic assessment

Mice skin samples were paraffin-embedded, and sections (5 μm in thickness) were stained with hematoxylin and eosin (HE). Dermal thickness was evaluated by measuring the distance between the epidermal-dermal junction and the dermal-fat junction in HE sections. Skin trichrome staining was performed by Masson’s trichrome stain kit (Polysciences, Warrington, PA, USA). The αSMA positive cells were counted in five random high-power fields using a light microscope. The mean score was used for analysis. The von Willebrand factor (vWF) staining intensity for immunohistochemical assessment was scored semi-quantitatively. The staining intensity (1: negative or weak staining, 2: moderate staining, and 3: strong staining) was evaluated in six randomly selected fields in the subcutaneous area. Then a semi-quantitative score per sample was generated by calculating the average of the six intensity scores per sample.

### Hydroxyproline assay

Collagen deposition was determined by measuring total hydroxyproline content in 8-mm skin punch biopsies obtained from PBS and Ang II infusion sites as described previously [16]. Briefly, the skin samples were hydrolyzed with 6 M sodium hydroxide at 120 °C for 16 h. The hydrolysate was then oxidized with oxidation buffer (one part 7% chloramine T and four parts acetate citrate buffer) for 4 minutes at room temperature. Ehrlich’s aldehyde reagent was added to each sample, and the chromophore was developed by incubating the samples at 60 °C for 25 minutes. Absorbance of each sample was read at 560 nm using a spectrophotometer. Results were expressed as relative hydroxyproline content. A standard curve was performed for all hydroxyproline measurements using known quantities of hydroxyproline.

### Immunohistochemical assessment

For single antibody staining, formalin-fixed, paraffin-embedded 5-μm skin tissue sections were de-paraffinized and rehydrated through a graded series of ethanol. Antigens were retrieved by incubation with a proteinase K solution (EMD Millipore, Billerica, MA, USA) for 5 minutes. Blocking was done by 2.5% normal horse serum for 1 h. Tissue sections were then incubated with primary antibody to αSMA (1:100, Novus Biologicals, Littleton, CO, USA), PDGFRβ (1:50, Cell Signaling Technology, Danvers, MA, USA), rat anti-mouse CD45 Ab (1:100, BD Pharmingen, San Diego, CA, USA), phospho-Smad2 (Ser465/467) Ab (1:100, Cell Signaling), or vWF (1:1000, DAKO, Santa Clara, CA, USA) at 4 °C for 16 h. Next, sections were incubated with ImmPress horseradish peroxidase (HRP) anti-rabbit IgG (Vector Laboratories, Burlingame, CA, USA) for 30 minutes. The color was developed using 3,3-diaminobenzidine (DAB) substrate (DAKO). Immunohistochemical images were collected using a microscope (BH-2; Olympus, Center Valley, PA, USA).

### Immunofluorescence staining

Staining was performed on 5-μm paraffin sections or cryosections. Slides were blocked with a blocking solution (3% BSA, and 0.2% Triton X-100 in PBS) for 2 h. Then, tissue sections were incubated at 4 °C overnight with primary antibodies: procollagen (*COL1A1* propeptide, 1:50, Thermo Fisher Scientific) for the cryosections and rabbit anti-mouse FSP1 (1:100, Abcam, Cambridge, MA, USA) for the paraffin sections. Secondary antibodies conjugated with Alexa 594 (Thermo Fisher Scientific) were used. Coverslips were mounted by using Vectashield with 4′,6-diamidino-2-phenylindole (DAPI) (Vector Laboratories, Burlingame, CA, USA). Fluorescence images were recorded with FV10i fluorescence microscope (Olympus, Tokyo, Japan).

### Statistical analysis

Values are presented as means ± standard deviation (SD). One-way analysis of variance with Tukey-Kramer test was used to determine significant differences between more than two groups. Analyses were performed with Statcel software (OMS, Tokorozawa, Japan). Significance was defined as *P* <0.05.

## Results

### Fibroblast-specific deletion of CTGF alleviates Ang II-induced skin fibrosis

**Fig. 1:**
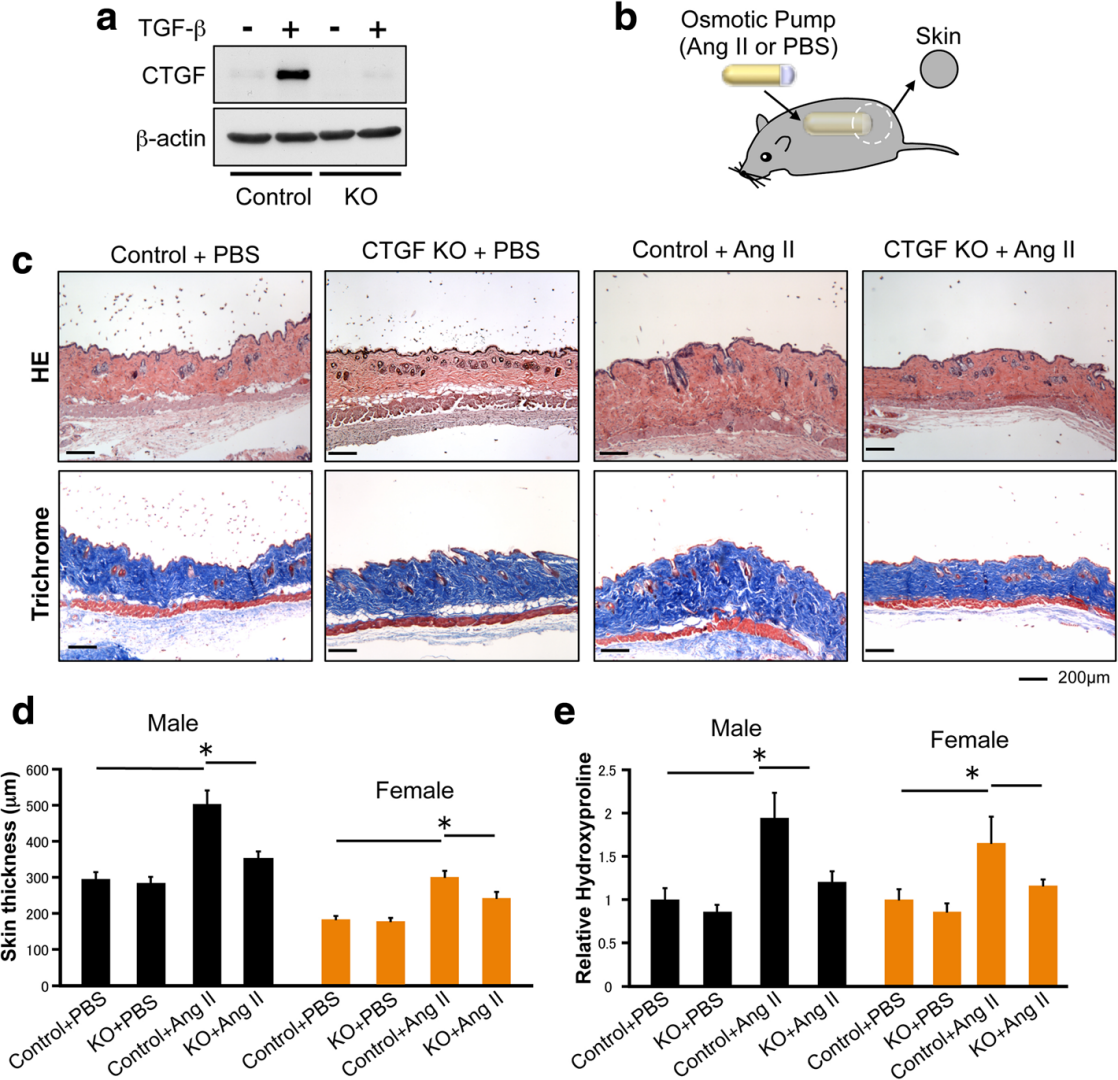
Fibroblast-specific connective tissue growth factor (*CTGF*) knockout inhibits angiotensin II (*Ang II*)-induced skin fibrosis. **a** Mice skin fibroblasts from CTGF knockout (*CTGF KO*) and control mice were treated for 24 h with transforming growth factor beta 1 (*TGF-*β*1*) (2.5 ng/ml). Cell lysates were subjected to immunoblotting. **b** Skin fibrosis in mice was induced by Ang II administered via subcutaneously installed osmotic pumps. After 2 weeks, the skin surrounding the pump outlet was collected. **c** Mice skin sections were stained with HE and trichrome. *Scale bar* 200 μm. **d** Dermal thickness is summarized. **e** Collagen contents were measured by hydroxyproline assay. Values are normalized relative to the PBS control group. Each graph represents mean ± SD; n = 3 per group; *P < 0.05

To evaluate the therapeutic effects of CTGF blockade in the in vivo model of SSc, we used a mouse model of Ang II-induced skin fibrosis [17]. Ang II-induced skin fibrosis is accompanied by diverse pathogenic mechanisms, including collagen accumulation, CTGF upregulation, myofibroblast accumulation, endothelial cell injury, inflammation, and fibrosis [15–17]. In an initial experiment, we examined the contribution of CTGF to Ang II-induced skin fibrosis using mice with smooth muscle cell fibroblast-specific deletion of CTGF (CTGF KO mice). We observed >80% reduction in CTGF protein levels in skin fibroblasts cultured from CTGF KO mice when compared to control mice (Fig. 1a).

**Fig. 2:**
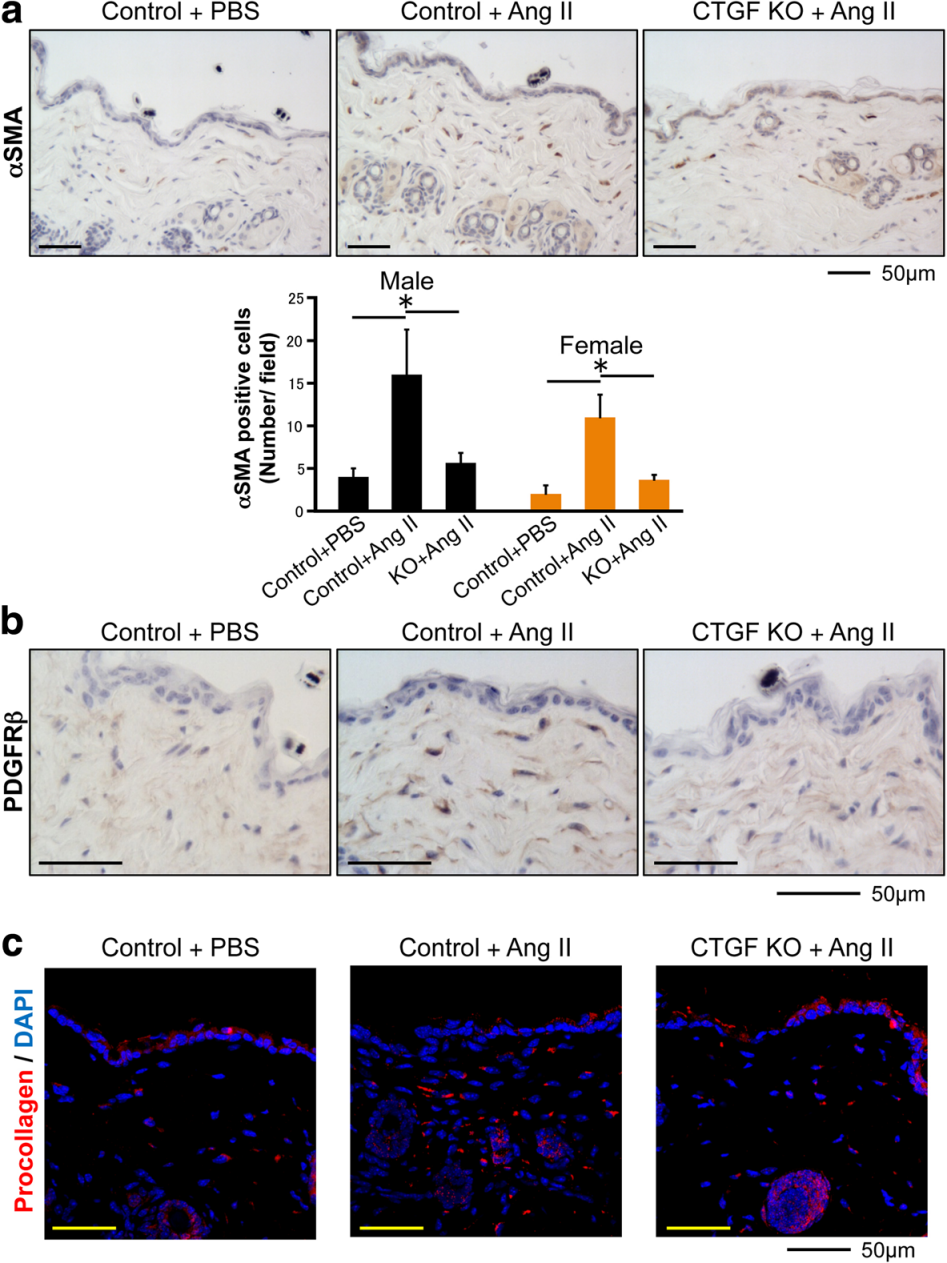
Fibroblast-specific connective tissue growth factor knockout (*CTGF KO*) reduces alpha smooth muscle actin (α*SMA*)-positive cells number. **a** Skin sections were stained with anti-αSMA antibody. Representative images are shown. The αSMA-positive cells were counted in five random high-power fields using a light microscope. The mean score was used for analysis. Each graph represents mean ± SD. **b** Skin sections were stained with anti-platelet-derived growth factor receptor β (*PDGFR*β) antibody. Representative images are shown. **c** Immunofluorescent staining was performed on cryosections using procollagen antibody. Representative images are shown. *Scale bar* 50 μm; n = 3 per group; *P < 0.05. *DAPI* 4′,6-diamidino-2-phenylindole

Because gender differences have been shown to affect pathogenic features, including the severity of fibrosis in SSc [23], we used both male and female mice in our experiments. CTGF KO and control male and female mice were then subjected to Ang II-induced skin fibrosis. Ang II or PBS was administered by subcutaneous osmotic pump, and the skin surrounding the pump outlet was collected on day 14 (Fig. 1b). In control mice, Ang II increased dermal thickness and the hydroxyproline content of lesional skin (Fig. 1c–e). Both male and female mice responded similarly to Ang II treatment. In contrast, male and female CTGF KO mice were resistant to the Ang II-induced changes (Fig. 1c–e). We next compared the number of αSMA-positive cells in the upper dermis of CTGF KO and control mice. The number of αSMA-positive cells in the upper dermis of CTGF KO mice challenged with Ang II was significantly decreased compared with control mice (Fig. 2a). Likewise, the number of PDGFRβ and procollagen positive cells were decreased in those mice (Fig. 2b and c). Collectively, these results indicated that CTGF is required for Ang II-induced skin fibrosis.

### FG-3019 attenuates Ang II-induced skin fibrosis

**Fig. 3:**
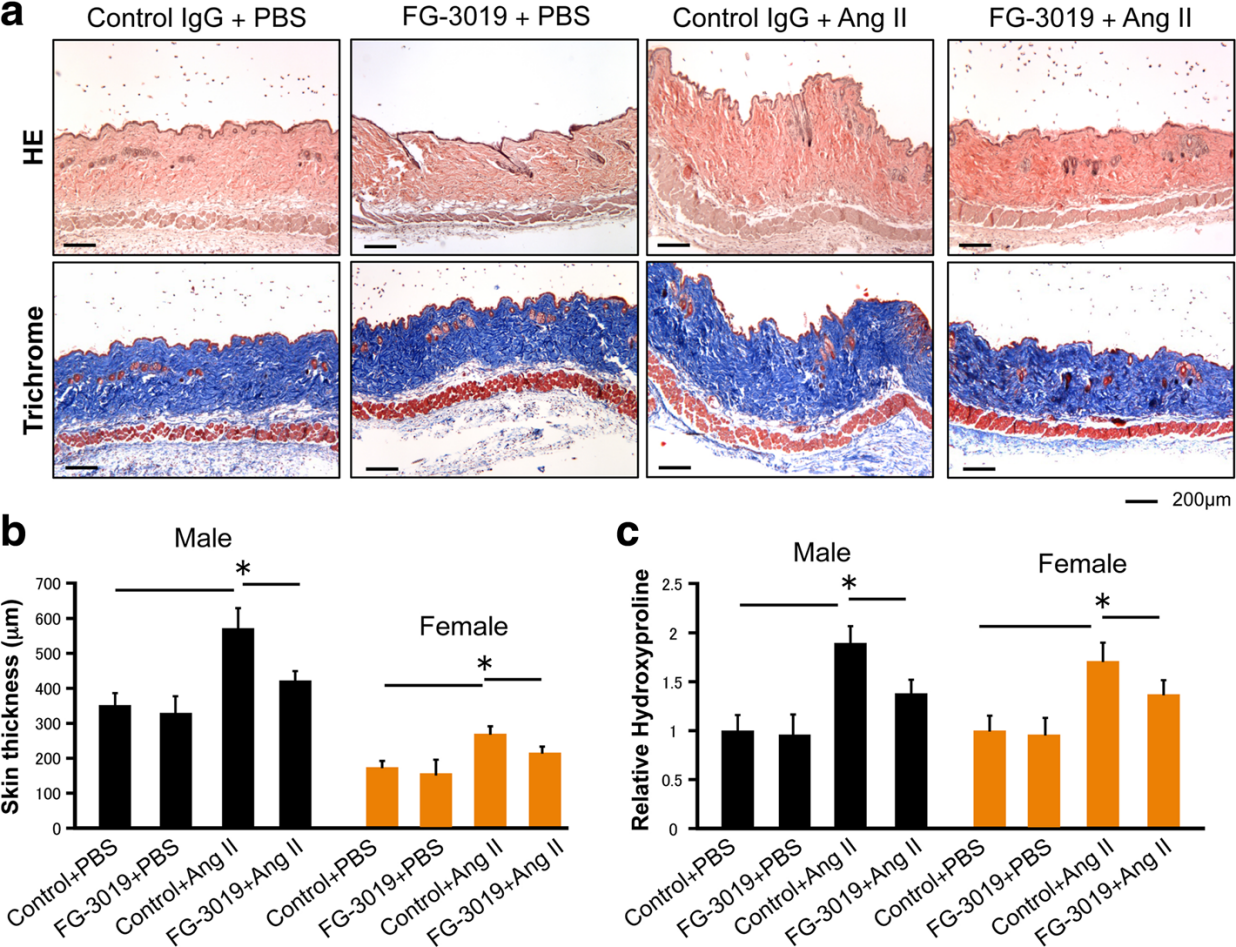
FG-3019 ameliorates angiotensin II (*Ang II*)-induced skin fibrosis. **a** Mice skin sections were stained with HE and trichrome. *Scale bar* 200 μm. **b** Dermal thickness is summarized. **c** Collagen contents were measured by hydroxyproline assay. Values are normalized relative to the PBS control group. Each graph represents mean ± SD; n = 4 per group; *P < 0.05

**Fig. 4:**
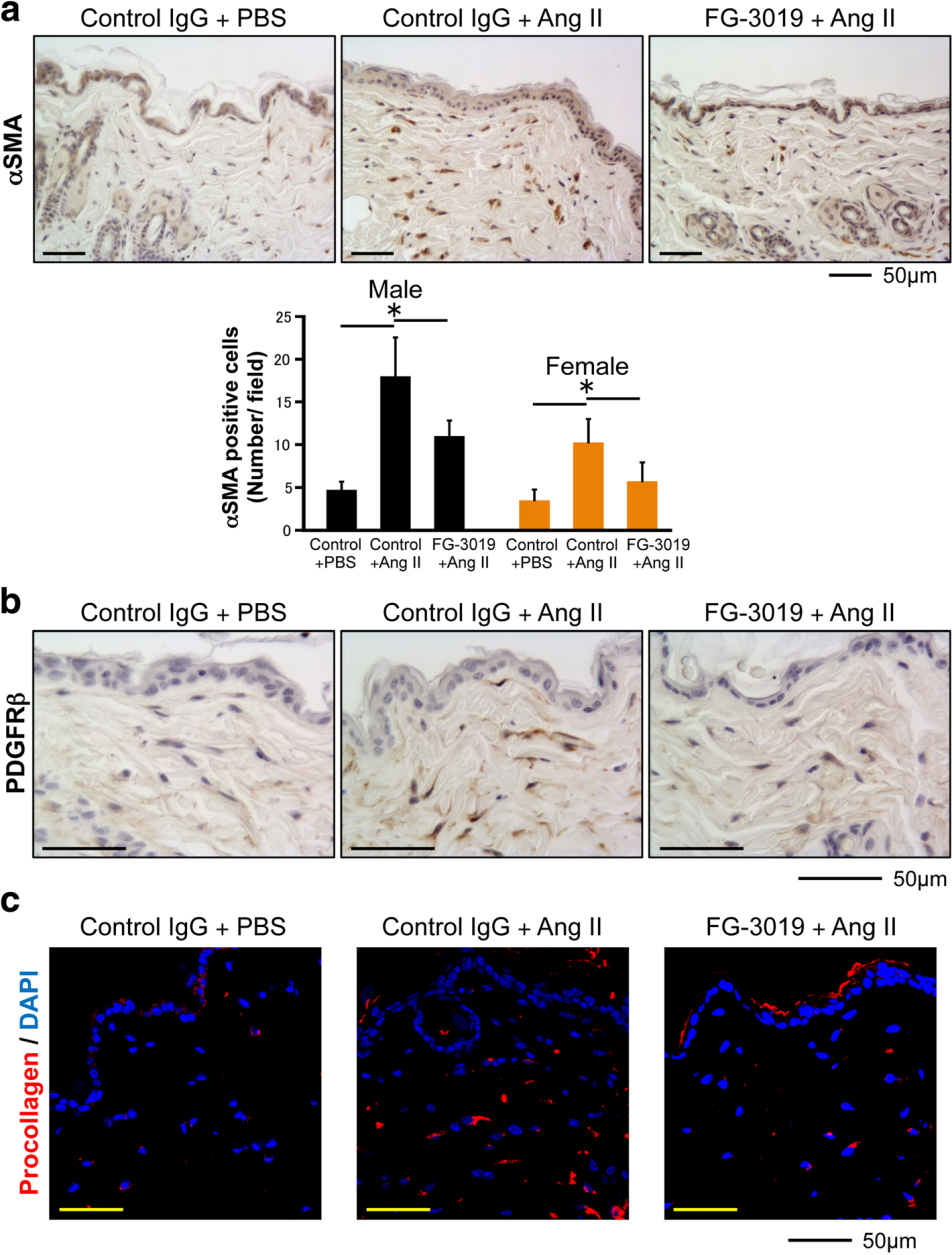
FG-3019 reduces alpha smooth muscle actin (α*SMA*)-positive cell numbers. **a** Skin sections were stained with anti-αSMA antibody. Representative images are shown. The αSMA-positive cells were counted in five random high-power fields using a light microscope. The mean score was used for analysis. Each graph represents mean ± SD. **b** Skin sections were stained with anti-platelet-derived growth factor receptor β (*PDGFR*β) antibody. Representative images are shown. **c** Immunofluorescent staining was performed on cryosections using procollagen antibody. Representative images are shown. *Scale bar* 50 μm; n = 4 per group; *P < 0.05. *DAPI* 4′,6-diamidino-2-phenylindole

We next investigated the effects of FG-3019 on Ang II-induced skin fibrosis. Ang II or PBS was administered by subcutaneous osmotic pump and FG-3019 (25 mg/kg) or control IgG (25 mg/kg) was administered intraperitoneally three times per week for 2 weeks. The skin surrounding the pump outlet was collected on day 14 (Fig. 3a). Treatment with FG-3019 significantly reduced dermal thickness and collagen content in skin from the backs of Ang II-challenged mice in both male and female animals (Fig. 3b and c). FG-3019 significantly decreased the number of αSMA-positive cells in the upper dermis of mice challenged with Ang II (Fig. 4a). FG-3019 also reduced PDGFRβ and procollagen expression in the upper dermis of mice challenged with Ang II (Fig. 4b and c). We only used male mice in subsequent experiments because we did not notice any apparent differences in responses to Ang II or the blockade of CTGF in male and female mice.

### Inhibition of CTGF ameliorates activation of TGF-β signaling in Ang II-induced skin fibrosis

**Fig. 5:**
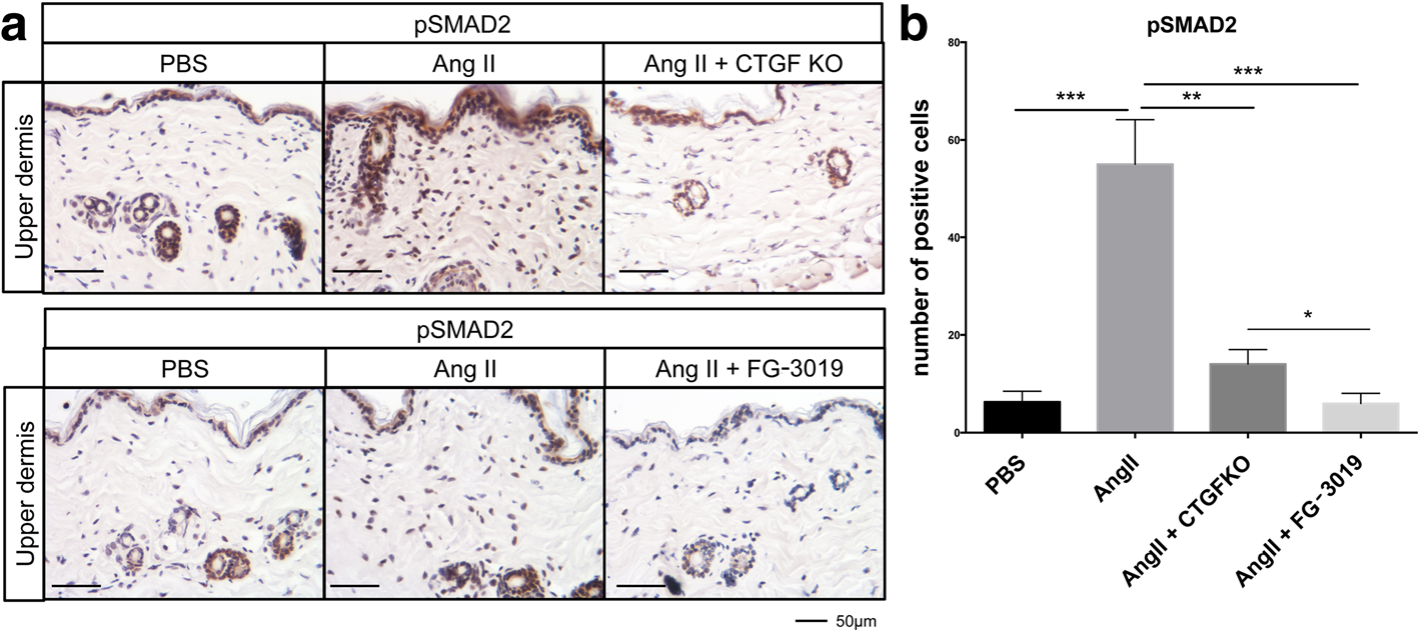
Blockade of connective tissue growth factor (*CTGF*) reduces the number of pSmad-positive cells. **a** Representative results of immunostaining for pSmad2. *Scale bar* 50 μm. **b** The pSmad2-positive cells were counted in five random high-power fields using a light microscope. The mean score was used for analysis. Each graph represents mean ± SD; *P < 0.05, **P < 0.01, ***P < 0.001. *Ang II* angiotensin II, *KO* knockout

We have previously shown activation of the TGF-β signaling pathway in the skin of mice challenged with Ang II [17]. As reported, Ang II induced a significant increase in pSmad2-positive cells distributed throughout the dermis. However, the number of pSmad2-positive cells was markedly reduced in CTGF KO mice (Fig. 5a). Interestingly, treatment with FG-3019 was significantly more effective than CTGF KO in reducing the number of pSmad2-positive cells comparable to the levels observed in control mice (Fig. 5b).

### Inhibition of CTGF reduces inflammation in the skin of Ang II treated mice

**Fig. 6:**
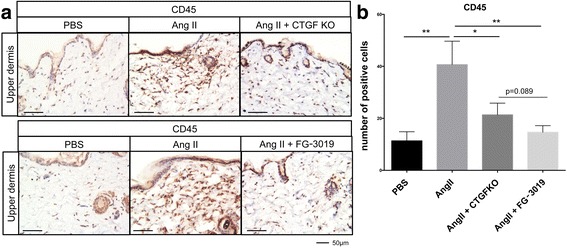
Blockade of connective tissue growth factor (*CTGF*) reduces the number of CD45-positive cells. **a** Representative results of immunostaining for CD45. *Scale bar* 50 μm. The CD45-positive cells were counted in five random high-power fields using a light microscope. The mean score was used for analysis. Each graph represents mean ± SD; *P < 0.05, **P < 0.01. *Ang II* angiotensin II, *KO* knockout

Ang II-induced skin fibrosis is accompanied by the increased presence of inflammatory cells in the dermis [17]. We next evaluated the effect of CTGF blockade on the recruitment of inflammatory cells. As shown in Fig. 6a, a significant increase in CD45-positive cells was observed in the skin of Ang II-challenged mice. The number of inflammatory cells was significantly reduced in the Ang II-challenged CTGF KO mice. Treatment with FG-3019 was somewhat more efficient in reducing the number of CD45-positive cells; however, the difference between the two treatments was not statistically significant (Fig. 6b).

**Fig. 7:**
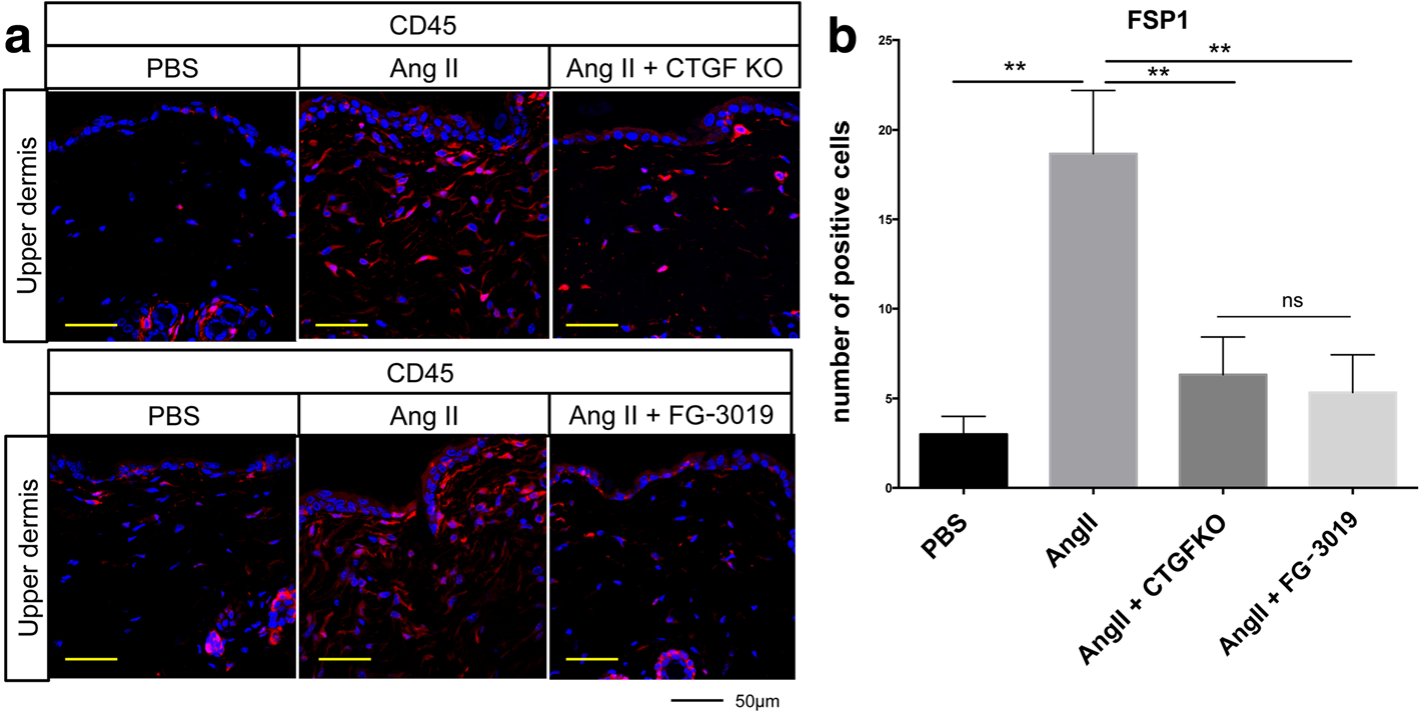
Blockade of connective tissue growth factor (*CTGF*) reduces the number of Fsp1 positive cells. **a** Representative results of immunostaining for Fsp1. *Scale bar* 50 μm. **b** The Fsp1-positive cells were counted in five random high-power fields using a fluorescent microscope. The mean score was used for analysis. Each graph represents mean ± SD; *P < 0.05, **P < 0.01. *Ang II* angiotensin II, *KO* knockout

We have previously shown that the Ang II model of skin fibrosis is characterized by the infiltration of fibrocytes (CD45^+^/FSP1^+^ double-positive cells) [17]. Consistent with these findings, immunofluorescent staining showed numerous FSP1-positive cells in the dermis of Ang II-infused mice, while inhibition of CTGF significantly reduced their number (Fig. 7a and b). Both treatments similarly reduced fibrocyte infiltration.

### Inhibition of CTGF reduces vascular damage in Ang II-induced skin fibrosis

**Fig. 8:**
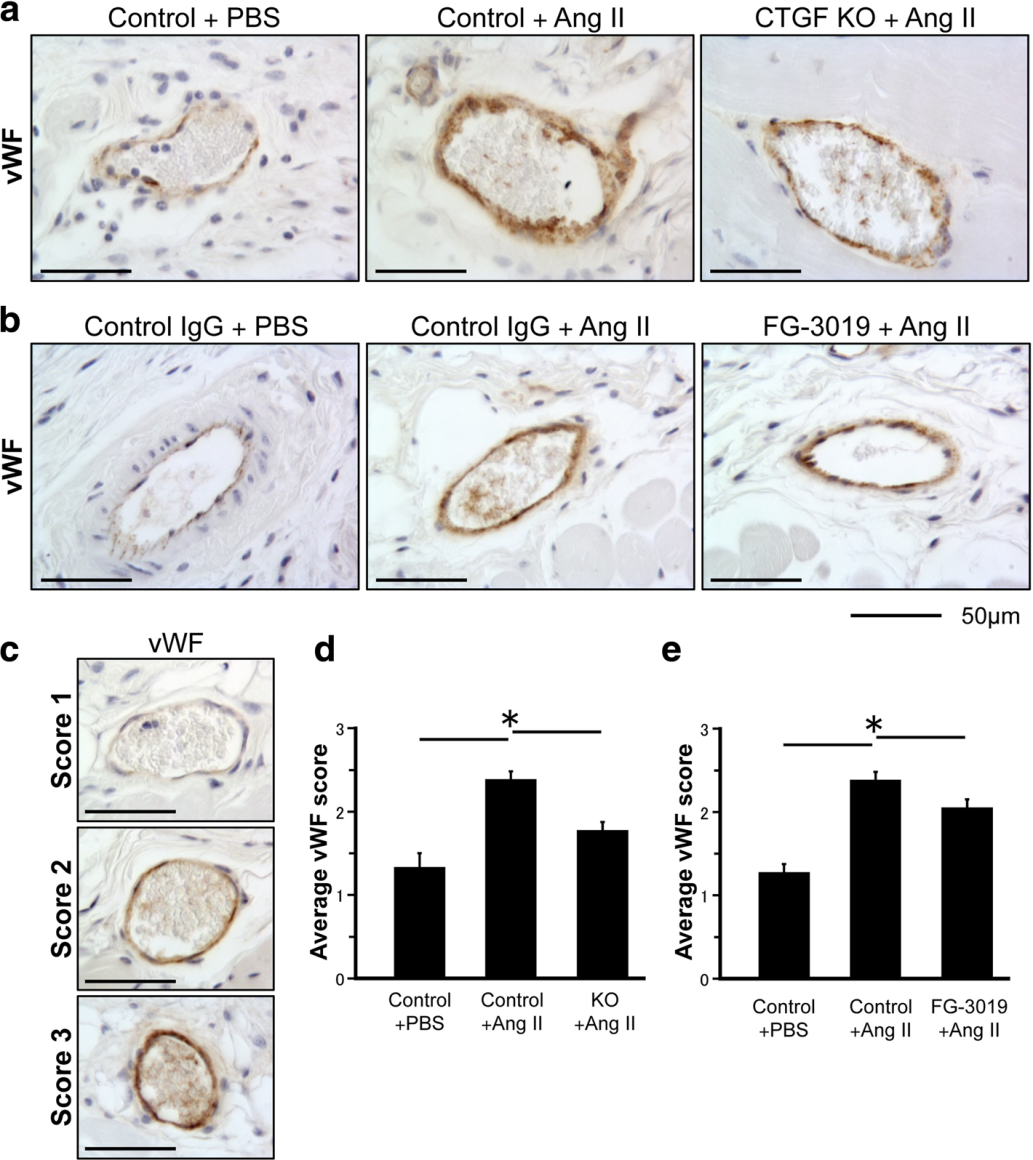
Inhibition of connective tissue growth factor (*CTGF*) ameliorates vascular damage in angiotensin II (*Ang II*)-induced fibrotic skin. **a**, **b** Representative results of immunostaining for von Willebrand factor (*vWF*). *Scale bar* 50 μm. **c** Representative staining intensity (1: negative or weak staining, 2: moderate staining, and 3: strong staining). **d**, **e** Semi-quantitative vWF vascular staining scores. Each graph represents mean score ± SD; n = 3 per group; *P < 0.05. *KO* knockout

Vascular damage is one of the key events in the pathogenesis of SSc. Mice challenged with Ang II develop prominent vascular injury [16]. We next examined whether inhibition of CTGF reduced vascular injury in this model. To evaluate the vascular injury, we used immunohistochemical staining of vWF, a widely used injury marker known to be elevated in SSc [24]. Consistent with the previous study [16], increased vWF staining was present in the dermal vessels of Ang II mice compared with the PBS control (Fig. 8a and b). The staining intensities were reduced in both CTGF KO and FG-3019-treated mice (Fig. 8a and b). Scoring of the staining intensity in the subcutaneous vessels and the representative images of each score (score 1–3) are shown in Fig. 8c. Although the intensity of vWF staining was significantly reduced in both CTGF KO and FG-3019-treated mice (Fig. 8d and e), the effect was more pronounced in CTGF KO mice.

## Discussion

Currently, there are only limited treatment options for patients with SSc. Recent studies have provided evidence of an important role of CTGF in the development of organ fibrosis, including the skin [25]. Blockade of CTGF using the human anti-CTGF antibody, FG-3019, effectively prevented the development of skin fibrosis in response to Ang II challenge. The anti-fibrotic effect of FG-3019 was comparable to the genetic deletion of CTGF in collagen-producing cells. In addition, both treatments were very effective in reducing inflammation and lessening the damaging effects of Ang II on skin microvasculature.

The current study supports the findings of Liu et al. [6] who demonstrated the essential role of CTGF in bleomycin-induced fibrosis. In both studies, deletion of CTGF from fibroblasts and smooth muscle cells/pericytes prevented increased collagen deposition and myofibroblast accumulation in the skin. In addition, we also showed that blockade of CTGF significantly reduced the number of PDGFRβ-positive cells in the fibrotic lesion. Although the origin of these PDGFRβ collagen-producing cells in this model is not known, these cells may represent the expansion of local fibroblasts. Alternatively, they could originate from pericytes or mesenchymal stem cells. The role of CTGF in recruitment and expansion of these cells is not clear at present. Side by side comparison of the anti-fibrotic effects of FG-3019 and genetic deletion of CTGF showed that antibody directed against CTGF was comparable to CTGF deletion in blocking the CTGF profibrotic function in this study. Our results are also consistent with previous findings that showed therapeutic effects of FG-3019 in bleomycin-induced lung fibrosis [26].

Although profibrotic functions of CTGF are most widely recognized, CTGF has also been shown to induce inflammatory response in various cell types in vitro [7]. The pro-inflammatory role of CTGF was further confirmed in in vivo models of pancreatic and renal inflammation [27, 28]. In our model of Ang II-induced fibrosis, which is also characterized by increased inflammation, blockade of CTGF almost completely abrogated infiltration of inflammatory cells, including fibrocytes. Interestingly, treatment with FG-3019 appeared to be more potent than genetic CTGF deletion in reducing infiltration of CD45-positive cells and pSmad2 cells. However, both treatments were similarly effective in reducing the number of fibrocytes. These findings are consistent with a recent report that showed an inhibitory effect of FG-3019 on acute and delayed immune responses in a model of radiation-induced pulmonary fibrosis [29], where the effect appears to be indirect via modulation of myofibroblast activation and chemokine secretion (K. Lipson, personal communication). Thus, in this study, it is conceivable that blockade of CTGF inhibits recruitment of inflammatory cells from the circulation either by preventing secretion of the pro-inflammatory chemokines/cytokines by the activated resident cells or by reducing vascular injury and vessel permeability.

Ang II has a detrimental effect on the vasculature by affecting both endothelial and vascular smooth muscle cells (vSMCs) [30]. CTGF plays an important role in the activation of vSMCs by promoting their growth, migration, and production of collagen. Furthermore, Ang II is known to induce CTGF in vSMCs [8]. It has also been reported that CTGF is overexpressed in microvascular endothelial cells in SSc and that conditioned medium from microvascular endothelial cells stimulates the proliferation and migration of fibroblasts in SSc [31]. Although the association between CTGF and cardiovascular diseases is well-documented [8, 32], less is known about the potential contribution of CTGF to vascular disease in SSc. In this study, we performed a limited assessment of the effects of CTGF blockade on vascular damage by measuring the vascular injury marker, vWF. We showed that inhibition of CTGF partially reduced vWF expression. The effect was more pronounced in the CTGF KO than in FG-3019-treated mice. Together, these data suggest that blockade of CTGF may have a beneficial effect on vascular injury. The limitation of this study is that it only assessed the preventive effect of FG-3019, and further studies are needed to evaluate whether FG-3019 is effective in reversing the established fibrosis. A phase II clinical trial evaluating FG-3019 as a treatment for idiopathic pulmonary fibrosis has recently been completed. Considering the anti-fibrotic effects of FG-3019 in the skin, our study strongly supports the testing of FG-3019 as a therapeutic agent for SSc dermal fibrosis.

## Conclusions

We showed that FG-3019 is effective in reducing Ang II-induced inflammation and skin fibrosis in mice. FG-3019 also partially reduced the Ang II-induced vascular damage. This study supports the testing of FG-3019 as therapy for skin fibrosis in patients with SSc.

## Abbreviations

Ang II: Angiotensin II
BSA: Bovine serum albumin
CTGF: Connective tissue growth factor
DAPI: 4′,6-Diamidino-2-phenylindole
ECM: Extracellular matrix
HE: Hematoxylin and eosin
KO: Knockout
PBS: Phosphate-buffered saline
PDGFRβ: Platelet-derived growth factor receptor β
SSc: Systemic sclerosis
vSMCs: vascular smooth muscle cells
vWF: von Willebrand factor
αSMA: Alpha smooth muscle actin
